# Elevated C-Reactive Protein as a Potential Biomarker for Neurological Adverse Events in Immune Checkpoint Inhibitor Therapy: A Prospective Cohort Study

**DOI:** 10.32604/or.2026.074095

**Published:** 2026-04-22

**Authors:** Laura Duzzi, Nora Möhn, Emily Narten, Janin Thomas, Susann Mahjoub, Lea Grote-Levi, Konstantin Jendretzky, Sandra Nay, Felix Konen, Jonas Wiegmann, Gernot Beutel, Tabea Fröhlich, Benjamin-Alexander Bollmann, Thomas Wirth, Imke von Wasielewski, Florian H. Heidel, Ralf Gutzmer, Thomas Skripuletz, Philipp Ivanyi, the ICOG (Immune Cooperative Oncology Group)-Investigators

**Affiliations:** 1 Department of Neurology, Hannover Medical School, Hannover, Germany; 2 Department of Haematology, Haemostaseology, Oncology and Stem Cell Transplantation, Hannover Medical School, Hannover, Germany; 3 Department of Pneumology, Hannover Medical School, Hannover, Germany; 4 Department of Gastroenterology, Hannover Medical School, Hannover, Germany; 5 Skin-Cancer-Center, Department of Dermatology, Allergology, and Venereology, Hannover Medical School, Carl-Neuberg-Str. 1, Hannover, Germany; 6 Department of Dermatology, Johannes Wesling Medical Center, Ruhr University Bochum, Minden, Germany; 7 Study Group, Comprehensive Cancer Center Niedersachsen (CCCN), Niedersachsen, Germany

**Keywords:** Immune checkpoint inhibitors (ICI), neurological adverse events, prospective cohort study, predictive biomarkers

## Abstract

**Objectives:**

Since 2011, immune checkpoint inhibitors (ICI) have transformed the treatment of various cancers. However, our understanding of the autoimmune adverse events, particularly those affecting the nervous system, remains limited. These adverse events can cause significant disability or even death, yet there are currently no established guidelines or biomarkers to aid diagnosis and treatment. With this study, we aim to gain a deeper understanding of neurological adverse events and investigate potential predictive biomarkers.

**Methods:**

Between 19 December 2019 and 21 August 2021, 150 out of 543 ICI-treated cancer patients were eligible for our prospective monocentric cohort study. Neurological assessments, clinical scores and the severity of side effects were analysed. Blood samples were taken before, during and after therapy. Patients with neurological AEs (the nAE group) and those without (the non-nAE group) were compared to identify potential predictive markers.

**Results:**

Of the 150 patients, 55 (36.7%) experienced nAE of any kind or severity, ranging from non-specific neurological symptoms to severe events. Severe nAE (Grade ≥ 3) was observed in 3.3% of patients and included cases of encephalitis and cerebral vasculitis. Regarding potential biomarkers, an increase in C-reactive protein (CRP) within the first 3–4 weeks was statistically associated with an increased likelihood of nAE in this study. As for patient- and treatment-related parameters, concurrent chemotherapy was found to be significantly associated with the occurrence of nAE.

**Conclusions:**

This study observed a relatively high rate of nAE under ICI therapy, partly due to the intentionally broad case definition. CRP elevation emerged as a potential predictive biomarker, warranting further investigation. However, other statistically significant markers did not consistently demonstrate clinical relevance.

## 1 Introduction

Since their introduction in 2011, immune checkpoint inhibitors (ICI) have changed the landscape of oncology and have become an integral part of cancer treatment [1]. While ICI were initially used primarily to treat patients with malignant melanoma, they are now a mainstay of therapy for various tumour types, including bronchial carcinoma, urothelial carcinoma, renal cell carcinoma, head and neck cancer, and squamous cell carcinoma of the skin, as well as other tumour types in gastroenterology, gynaecology and haematology [2].

Alongside chemotherapy and radiotherapy, ICI represent one of the main pillars of cancer treatment [3]. As the name suggests, they inhibit immune checkpoints, which are a built-in system of the human body that suppresses T cell activity in order to prevent the immune system from overreacting. However, tumour cells, due to cancer cell evolution, can activate immune checkpoints and therefore exploit these pathways to evade T cell toxicity. Through their mechanism of action, ICI improve the immune system’s anti-tumour response and increase T cell toxicity against tumour cells. Typically, ICI target one of the two most well-characterised immune checkpoints: cytotoxic T-lymphocyte-associated protein 4 (CTLA-4) and programmed cell death protein 1 (PD-1), or its ligand PD-L1 [4]. While ICI can provide significant benefits for patients with cancer [5], the T cell activation induced by this therapeutic approach can lead to a new class of adverse events known as immune-related adverse events (irAEs). These events are based on autoreactivity and can resemble autoimmune diseases. Depending on their severity, irAE can limit therapy or even be life-threatening [6]. ICI can induce irAEs across nearly all organ systems. While most patients experience toxicity confined to one system, combined inhibition of CTLA-4 and PD-1 substantially heightens the risk of multisystem manifestations [7]. The most commonly affected organs are the skin, lungs, gastrointestinal tract, and endocrine organs [8]. The corresponding clinical manifestations include mucositis, vitiligo, colitis, hepatitis, thyroid dysfunction and hypophysitis. The incidence and severity of these irAEs vary depending on the specific ICI regimen, as well as the underlying malignancy [9].

This study examines a specific subset of irAEs targeting the nervous system, referred to as neurological adverse events (nAE). While less common than other immune-related toxicities, ICI-induced neurotoxicity can result in profound and occasionally persistent functional deficits and may be fatal [10]. Compared with other adverse events, certain nAE exhibit a particularly high mortality risk [11]. These include autoimmune encephalitis as well as myasthenia-myositis overlap syndromes [12]. Recent studies have reported the incidence of nAE associated with ICI to range from 0.24% to 12%, depending on the type of ICI used [13–15]. Severe nAE, classified as grade 3 or higher according to the Common Terminology Criteria for Adverse Events (CTCAE), seem to occur in less than 1% of cases [11]. However, emerging real-world data suggest that these published findings likely underestimate the true incidence [16].

When nAE occur during ICI therapy, early initiation of anti-inflammatory treatment is crucial to improving patient outcomes [17–19]. Yet diagnosing nAE remains challenging in routine clinical practice due to the limited understanding of their pathophysiology and the fact that many oncology teams have comparatively little experience in managing neurological complications. In addition, published data on the clinical presentation, management, and prognosis of nAE vary considerably [17].

The aim of this study is to deepen the understanding of nAE by characterizing their incidence, clinical features, and diagnostic markers. Because the occurrence of nAE cannot currently be predicted before the start of ICI therapy, identifying potential pre-therapeutic biomarkers is of particular interest. To address this unmet need, we conducted a prospective monocentric cohort study to explore biomarkers and clinical risk factors that may predict the development of nAE during ICI treatment.

## 2 Material and Methods

### 2.1 Study Design and Patient Selection

Our data were collected as part of a prospective, interdisciplinary ICOG (Immune Cooperative Oncology Group) cohort study initiated in November 2019 at Hannover Medical School (MHH). This study was conducted in collaboration with the Skin Cancer Centre and the Departments of Haematology, Haemostasis, Oncology and Stem Cell Transplantation; Pneumology; Gastroenterology. The study’s primary aim was to monitor patients receiving ICI therapy before and during treatment to identify nAE at an early stage and gather data on potential biomarkers. Of the 543 ICI-treated cancer patients enrolled over a 27-month period, 154 were included in this analysis, with four cases excluded due to protocol violations. All adult cancer patients (≥18 years) receiving ICI-therapy who provided written informed consent were eligible. Inclusion required completion of the baseline visit prior to treatment initiation. Patients were excluded if they had already received the first dose of ICI prior to enrollment in the study or if they did not provide written informed consent. The comparatively low inclusion proportion reflects the phased implementation of the study infrastructure in the early months and the requirement for baseline sampling prior to the first ICI dose. Patients who had already initiated treatment before enrolment could not be included retrospectively. The cohort comprised ICI-treated oncological patients with various tumour types (Table 1). During the six-month study period, patients underwent a series of predefined assessments starting prior to treatment initiation and followed by visits at three- to four-week intervals (Supplementary Table S1). As the observation protocol was expanded during the study, not all patients completed all seven visits. The study protocol was approved by the local ethics committee at Hannover Medical School (No. 8685_BO_K2019) following the Declaration of Helsinki. Informed consent was obtained from all individual participants included in the study. Patients signed informed consent regarding publishing their data.

**Table 1:** Disease characteristics of the overall cohort and the subgroups with and without neurotoxicity.

Parameter	Total Cohort (n = 150)	Non-nAE-Subgroup (n = 95)	nAE-Subgroup (n = 55)	*p*-value
**Age at first diagnosis**, years, Median (range)	60 (25–85)	62 (25–85)	58 (28–80)	0.098
**Sex**				
Female, n (%)	54 (36%)	35 (36.8%)	19 (34.5%)	0.778
Male, n (%)	96 (64%)	60 (63.2%)	36 (65.5%)	0.778
**Underlying tumor entity**				
Malignant melanoma, n (%)	98 (65.3%)	72 (75.8%)	26 (47.3%)	**<0.001**
Renal cell carcinoma, n (%)	18 (12.0%)	10 (10.5%)	8 (14.5%)	0.465
NSCLC, n (%)	13 (8.7%)	5 (5.3%)	8 (14.5%)	0.052
HNSCC, n (%)	12 (8.0%)	5 (5.3%)	7 (12.7%)	0.023
HCC, n (%)	3 (2.0%)	1 (1.1%)	2 (3.6%)	0.276
Cutaneous SCC, n (%)	2 (1.3%)	1 (1.1%)	1 (1.8%)	0.694
SCLC, n (%)	2 (1.3%)	0 (0.0%)	2 (3.6%)	0.061
Pleura mesothelioma, n (%)	1 (0.7%)	1 (1.1%)	0 (0.0%)	0.455
CUP, n (%)	1 (0.7%)	0 (0.0%)	1 (1.8%)	0.187

Note: Significant differences between nAE- and non-nAE-subgroup are printed in bold. nAE: neurological adverse events, CUP: cancer of unknown primary, HCC: hepatocellular carcinoma, HNSCC: head and neck squamous cell carcinoma, NSCLC: non-small cell lung cancer, SCC: squamous cell carcinoma, SCLC: small cell lung cancer.

### 2.2 Neurological Symptoms Assessment

The subinvestigators received standardized training in clinical neurological examination provided by board-certified neurologists. The training focused on harmonizing examination techniques and documentation to ensure consistent neurological assessments across investigators. If any neurological abnormalities were detected during a visit or if a patient reported new symptoms, the attending physicians promptly initiated the necessary diagnostic and therapeutic measures. For the purposes of this study, all symptoms involving the nervous system were meticulously documented. This encompassed impairments of the central and peripheral nervous system, neuromuscular involvement, and other neurological symptoms, as long as no alternative etiologies were identified. Alternative causes were excluded through standardized neurological evaluation and targeted diagnostic workup performed by experienced neurologists. Any exacerbations of pre-existing neurological conditions were also carefully recorded to ensure a comprehensive approach. All symptoms aligning with these categories were considered nAE within the scope of this study.

### 2.3 Demographic and Diagnostic Data

Data acquisition was carried out by the responsible data manager. The collected data included baseline characteristics assessed prior to initiation of ICI therapy including age, sex, tumour type, cerebral metastasis, TNM (tumor, node, metastasis) stage, PD-L1 status and relevant comorbidities categorised as cardiovascular, pulmonary or autoimmune diseases. TNM stage was determined according to standard clinical and radiological staging procedures, and PD-L1 status was assessed using routine immunohistochemical analyses performed as part of standard pathological evaluation. Lifestyle factors such as nicotine and alcohol use were documented, alongside detailed treatment-related information, including ICI type, previous cancer therapies, and any concomitant oncological treatments. IrAEs were systematically recorded and graded according to the Common Terminology Criteria for Adverse Events (CTCAE, version 5.0). Treatment impact was operationally defined as any irAE leading to treatment discontinuation, temporary treatment interruption, dose delay, or hospitalisation., nAE were assessed according to established neurological syndromes and their clinical severity. No dedicated nAE scoring system was used. Assessments were based on standardized neurological evaluations and documentation. Supporting data were extracted from either digital or analogue patient records at Hannover Medical School. Although record formats differed across institutions, data extraction was performed according to standardized protocols using predefined variables, thereby ensuring consistency and comparability between digital and analogue sources.

### 2.4 Blood Samples

Blood samples (serum and plasma) were collected from all patients at each study visit under standardized conditions according to institutional protocols, including predefined collection times, standardized processing, and immediate transfer to the central laboratory. A comprehensive panel of 36 laboratory parameters was analysed using standardised procedures to ensure a broad and inclusive approach. All laboratory analyses were performed at the accredited central laboratory of Hannover Medical School (DIN EN ISO 15189) using standardized automated platforms. Clinical chemistry parameters were measured using photometric or enzymatic assays, immunological markers using electrochemiluminescence-based immunoassays, and blood counts using automated hematology analyzers based on flow cytometry and impedance methods. The parameters included electrolytes (potassium, sodium, calcium and magnesium); inflammatory markers (C-reactive protein [CRP] and soluble interleukin-2 receptor [sIL-2R]); growth differentiation factor 15 (GDF-15); kidney function markers (creatinine and urea); cardiac markers (troponin T and N-terminal pro-B-type natriuretic peptide [NT-proBNP]); liver enzymes (aspartate aminotransferase [AST], alanine aminotransferase [ALT], gamma-glutamyl transferase [GGT], bilirubin, albumin and lactate dehydrogenase [LDH]); and metabolic markers (lipase, glucose and ferritin). Blood counts (haemoglobin, platelets and leukocytes, including neutrophils, eosinophils and lymphocytes), coagulation factors (prothrombin time), cortisol levels and thyroid function markers (free thyroxine [fT4], free triiodothyronine [fT3] and thyroid-stimulating hormone [TSH]) were also measured.

### 2.5 Statistics

All data were consolidated into a Microsoft Excel database (2010), which was then checked for completeness and plausibility. Statistical analyses were conducted using IBM SPSS Statistics (version 28.0.1.0, IBM Corp., Armonk, NY, USA), setting significance at *p* < 0.05. The cohort was divided into two groups: those who experienced nAE (the nAE subgroup) and those who did not (the non-nAE subgroup). Descriptive data analysis was performed, including the calculation of medians, ranges (minimum and maximum) and relative frequencies. Subgroup comparisons were carried out using the appropriate statistical tests, including Student’s *t*-test, Mann–Whitney U test and chi-squared test, depending on the variable type. Variables with a *p*-value of less than 0.2 were analysed further using univariate binary logistic regression.

Laboratory analyses focused on whether deviations above or below the cohort median in selected parameters were associated with nAE. To reduce model complexity, parameters were dichotomised at the median rather than analysed as continuous values. Those showing significantly higher levels in the nAE group (unpaired *t*-test) were further assessed as potential risk factors in binary logistic regression. Again, a cutoff of *p* < 0.2 was applied.

Lastly, three multivariate models were created for further analyses.

1. Patient characteristics: age at first diagnosis; malignant melanoma (yes/no); NSCLC (yes/no); metastasis (yes/no)
2. Treatment characteristics: age at start of ICI; PD-(L)-1-inhibitor + chemotherapy (yes/no)
3. Laboratory examinations:
  a. FU1: CRP; LDH; bilirubin
  b. FU3: GGT; ferritin; fT4
  c. FU4: urea; platelets
  d. FU5: creatinine; AST; platelets; neutrophils; prothrombin time
  e. EOS: CRP; LDH; albumin

For each time point, separate models were generated for the respective laboratory parameters. The results of the multivariate analyses were deemed significant at *p* < 0.05.

## 3 Results

### 3.1 Patients’ Characteristics

Between December 2019 and August 2021, 150 patients undergoing ICI treatment were included in the study. IrAEs occurred in 104 of these patients (69.3%). Of these, 55 patients (36.7%) experienced nAE of varying severity. Neurotoxicity accounted for 26.4% of all irAEs documented in this cohort. The median age of the cohort was 63 years (range: 29–87), and 36% of patients were female. There were no significant differences in baseline characteristics between the nAE and non-nAE groups (Table 1).

The majority of patients was treated for malignant melanoma (65.3%), renal cell carcinoma (12%), or non-small cell lung cancer (NSCLC) (8.7%). Interestingly, malignant melanoma was less frequent among patients who developed nAE compared to those without nAE (47.3% vs. 75.8%, *p* < 0.001), while a higher proportion of patients in the nAE group had NSCLC (14.5% vs. 5.3%, *p* = 0.052).

Regarding ICI therapy, nivolumab was the most commonly used agent (50.7%), followed by nivolumab/ipilimumab (21.3%) and pembrolizumab (14.7%). Significantly fewer patients in the nAE group received nivolumab (34.5% vs. 60.0%, *p* = 0.003), whereas pembrolizumab was more frequently administered in this group (25.5% vs. 8.4%, *p* = 0.004). In addition, ICI-therapy combined with chemotherapy was more commonly used among patients who developed nAE (21.8% vs. 5.3%, *p* = 0.002). The median time between initial tumour diagnosis and ICI initiation was 9.5 months (range: 0–243). A higher proportion of patients in the nAE group received a palliative treatment regime at the start of therapy (nAE: 72.7% vs. non-AE: 53.7%, *p* = 0.021) (Table 2).

**Table 2:** Characteristics of therapy of the overall cohort and the subgroups with and without neurotoxicity.

Parameter	Total Cohort (n = 150)	Non-nAE-Subgroup (n = 95)	nAE-Subgroup (n = 55)	*p*-Value
**Age at start of ICI**, years, Median (range)	63 (29–87)	64 (29–86)	62 (29–87)	0.137
**ICI**				
Nivolumab, n (%)	76 (50.7%)	57 (60.0%)	19 (34.5%)	**0.003**
Nivolumab/Ipilimumab, n (%)	32 (21.3%)	20 (21.1%)	12 (21.8%)	0.912
Pembrolizumab, n (%)	22 (14.7%)	8 (8.4%)	14 (25.5%)	**0.004**
Atezolizumab, n (%)	8 (5.3%)	3 (3.2%)	5 (9.1%)	0.119
Durvalumab, n (%)	2 (1.3%)	0 (0.0%)	2 (3.6%)	0.061
Cemiplimab, n (%)	2 (1.3%)	1 (1.1%)	1 (1.8%)	0.694
Ipilimumab, n (%)	1 (0.7%)	1 (1.1%)	0 (0.0%)	0.445
**PD(L)1-Inh. and TKI, n (%)**	7 (4.7%)	5 (5.3%)	2 (3.6%)	0.649
**Setting**				
Palliative, n (%)	91 (60.7%)	51 (53.7%)	40 (72.7%)	**0.021**
Adjuvant, n (%)	59 (39.3%)	44 (46.3%)	15 (27.3%)	**0.021**
**CTX in addition to ICI, n (%)**	17 (11.3%)	5 (5.3%)	12 (21.8%)	**0.002**

Note: Significant differences between nAE- and non-nAE-subgroup are printed in bold. Legend: nAE: neurological adverse events, ICI: immune checkpoint inhibitors, CTX: chemotherapy, PD-1: programmed cell death protein 1, TKI: tyrosine kinase inhibitor.

### 3.2 Characterization of all Immune Related Adverse Events

In this study, both nAE and irAEs affecting other organ systems were systematically monitored. Overall, approximately two-thirds of patients experienced at least one irAE, and 30.7% developed more than one (Table 3). The first irAE occurred on average 32 days after therapy initiation. ICI treatment was discontinued in 15.3% of patients (n = 23), and 7.3% (n = 4) required hospitalization. Beyond nAE, the most frequent severe irAEs (CTCAE ≥ 3) involved the gastrointestinal tract (12%). The most common irAEs overall were thyroiditis/hyperthyroidism (9.3%), colitis (8%), and hepatitis (5.3%). Notably, gastrointestinal irAEs differed significantly between groups: patients with nAE experienced fewer severe gastrointestinal irAEs (3.6% vs. 16.8%, *p* = 0.016), and none developed colitis. The distribution of other specific irAEs was comparable between groups (Table 3).

**Table 3:** Characteristics of all immune related adverse events (irAE) within the whole cohort and both subgroups.

Parameter	Total Cohort (n = 150)	Non-nAE-Subgroup (n = 95)	nAE-Subgroup (n = 55)	*p*-Value
**Number of irAEs per patient**				
0 irAE, n (%)	46 (30.7%)	30 (31.6%)	16 (29.1%)	0.503
1 irAE, n (%)	58 (38.7%)	38 (40.0%)	20 (36.4%)	0.503
2 irAE, n (%)	25 (16.7%)	15 (15.8%)	10 (18.2%)	0.503
≥3 irAE, n (%)	21 (14.0%)	12 (12.6%)	9 (16.4%)	0.503
**Time until onset of irAEs** Median (Range), days	32 (0–202)	28 (0–186)	35 (0–202)	0.786
**Overview of all irAEs, n (%)**				
**Overall**				
nAE	55 (36.7%)	0 (0.0%)	55 (100%)	/
Skin	47 (31.3%)	29 (30.5%)	18 (32.7%)	0.779
Gastrointestinal tract	41 (27.3%)	27 (18.4%)	14 (25.5%)	0.694
Endocrine system	19 (12.7%)	14 (14.7%)	5 (9.1%)	0.316
Joints	9 (6.0%)	4 (4.2%)	5 (9.1%)	0.225
Respiratory tract	9 (6.0%)	6 (6.3%)	3 (5.5%)	0.831
Cardiovascular system	4 (2.7%)	3 (3.2%)	1 (1.8%)	0.624
Others*	24 (16%)	11 (11.6%)	13 (23.6%)	0.052
**CTCAE ≥ 3**				
Gastrointestinal tract	18 (12.0%)	16 (16.8%)	2 (3.6%)	**0.016**
nAE	5 (3.3%)	0 (0.0%)	5 (9.1%)	/
Endocrine system	5 (3.3%)	4 (4.2%)	1 (1.8%)	0.432
Joints	4 (2.7%)	1 (1.1%)	3 (5.5%)	0.107
Skin	3 (2.0%)	2 (2.1%)	1 (1.8%)	0.904
Respiratory tract	0 (0.0%)	0 (0.0%)	0 (0.0%)	/
Cardiovascular system	0 (0.0%)	0 (0.0%)	0 (0.0%)	/
Others*	4 (2.7%)	3 (3.2%)	1 (1.8%)	0.624
**Specific irAEs other than nAE**				
Thyroiditis/Hyperthyroidism, n (%)	14 (9.3%)	10 (10.5%)	4 (7.3%)	0.509
Colitis, n (%)	12 (8.0%)	12 (12.6%)	0 (0.0%)	**0.006**
Hepatitis, n (%)	8 (5.3%)	7 (7.4%)	1 (1.8%)	0.145
Nephritis/AKI, n (%)	5 (3.3%)	4 (4.2%)	1 (1.8%)	0.432
Dermatitis, n (%)	4 (2.7%)	1 (1.1%)	3 (5.5%)	0.107
Hypophysitis, n (%)	4 (2.7%)	4 (4.2%)	0 (0.0%)	0.123
Pancreatitis, n (%)	3 (2.0%)	2 (2.1%)	1 (1.8%)	0.904
Arthritis, n (%)	2 (1.3%)	1 (1.1%)	1 (1.8%)	0.694
Autoimmune thrombozytopenia, n (%)	1 (0.7%)	1 (1.1%)	0 (0.0%)	0.445
Hemophagocytic lymphohistiocytosis, n (%)	1 (0.7%)	1 (1.1%)	0 (0.0%)	0.445

Note: Significant differences between nAE- and non-nAE-subgroup are printed in bold. *Others include: ophthalmology, hematology, infectiology. nAE: neurological adverse events, ICI: immune checkpoint inhibitors, irAEs: immune related adverse events, CTCAE: common terminology criteria for adverse events, AKI: acute kidney injury.

### 3.3 Characterization of Neurological Adverse Events

In total, 55 patients (36.7%) experienced at least one nAE, with 9.1% (n = 5) developing more than one. The majority of nAE arose within six to eight weeks, with an average onset of 42 days after ICI initiation (Supplementary Fig. S1). Peripheral nAE were predominant, with polyneuropathy (40%) and paresthesia/hypesthesia (34.6%) being the most common, followed by myalgia, myositis and paresis (Table 4). Events affecting the central nervous system, such as cerebral vasculitis and encephalitis, were rare (1.8%). Most nAE were mild or moderate (CTCAE I–II in 90.9%), but 9.1% were severe (CTCAE III). ICI therapy was discontinued in 7.3% of cases and paused in 3.6%. Outpatient treatment was provided in 20% of cases and included corticosteroids (9.1%) and intravenous immunoglobulins. (1.3%). Four patients (7.3%) were hospitalised due to severe nAE, with cases including giant cell arteritis, polyneuropathy and autoimmune brainstem encephalitis. Only 23.6% of patients achieved full recovery within six months, while 7.3% showed partial improvement and 41.8% showed no improvement by the end of the study period. In 27.3% of cases, recovery status could not be reliably verified; these patients were therefore classified as lost to follow-up (Table 4).

**Table 4:** Characteristics of neurological adverse events (nAE).

Parameter	Total Cohort (n = 150)	nAE-Subgroup (n = 55)
**Specific nAE**		
Polyneuropathy, n (%)	22 (14.7%)	22 (40.0%)
Paresthesia/hypesthesia, n (%)	19 (12.7%)	19 (34.6%)
Myalgia, n (%)	7 (4.7%)	7 (12.7%)
Myositis/elevated CK levels, n (%)	5 (3.3%)	5 (9.1%)
Paresis, n (%)	2 (1.3%)	2 (3.6%)
Cerebral vasculitis, n (%)	1 (0.7%)	1 (1.8%)
Encephalitis, n (%)	1 (0.7%)	1 (1.8%)
Restless-Leg-Syndrome, n (%)	1 (0.7%)	1 (1.8%)
Abscence like states, n (%)	1 (0.7%)	1 (1.8%)
Tremor, n (%)	1 (0.7%)	1 (1.8%)
**Time until onset of nAE, days, Median (Range)**	42 (0–202)	42 (0–202)
**nAE CTCAE**		
CTCAE 1, n (%)	20 (13.3%)	20 (36.4%)
CTCAE 2, n (%)	30 (20.0%)	30 (54.5%)
CTCAE 3, n (%)	5 (3.3%)	5 (9.1%)
**Additional diagnostics’#**		
Electrophysiology, n (%)	6 (4.0%)	6 (10.9%)
Lumbar puncture, n (%)	4 (2.7%)	4 (7.3%)
MRI, n (%)	3 (2.0%)	3 (5.5%)
Other*, n (%)	6 (4.0%)	6 (10.9%)
**Management of nAE**		
Outpatient, n (%)	11 (7.3%)	11 (20.0%)
Hospitalization, n (%)	4 (2.7%)	4 (7.3%)
No therapy necessary, n (%)	40 (26.7%)	40 (72.7%)
**Treatment of nAE**		
Steroids, n (%)	5 (3.3%)	5 (9.1%)
IVIG, n (%)	1 (0.7%)	1(1.8%)
Other/symptomatic, n (%)	10 (6.7%)	10 (18.2%)
None, n (%)	40 (26.7%)	40 (72.7%)
**Outcome nAE**		
Complete Recovery, n (%)	13 (8.7%)	13 (23.6%)
Improvement, n (%)	4 (2.7%)	4 (7.3%)
No recovery, n (%)	23 (15.3%)	23 (41.8%)
N/A, n (%)	15 (10.0%)	15 (27.3%)

Note: CK: creatine kinase; CTCAE: Common Terminology Criteria for Adverse Events; MRI: magnetic resonance imaging; IVIG: intravenous immunoglobulin; N/A: not available. #: As written in material and methods, all patients received clinical neurological assessment, as well additional neurological diagnostics; percentages reflect those of additional diagnostic which defined nAE. *Other: vigorimeter, temporal artery biopsy, electrocardiogram, cranial computer tomography, ophthalmological examination.

### 3.4 Laboratory Examinations

**Figure 1:**
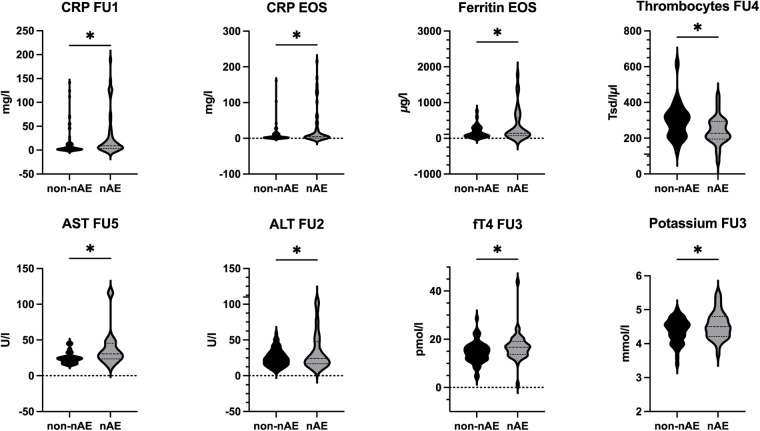
Illustration of serum parameters with significant differences between the nAE and non-nAE subgroups at different time points. Visualization as violin plot (mean + SD). EOS: end of study; FU: follow-up; nAE: neurological adverse events; CRP: C-reactive protein; AST: aspartate aminotransferase; ALT: alanine aminotransferase; fT4: free thyroxine. *p < 0.05.

Blood samples were collected at each follow-up to monitor standard laboratory parameters as well as additional markers. Significant differences between the nAE and non-nAE groups were observed at specific time points for potassium, CRP, AST, ALT, ferritin, platelets, and fT4 (Fig. 1, Supplementary Table S2).

### 3.5 Uni- and Multivariate Analysis

Subgroup differences discovered in the subsequent uni- and multivariate analyses were examined further and categorised into three groups: patient characteristics, treatment characteristics, and laboratory examinations. Univariate analysis confirmed a correlation for three patient and treatment characteristics (Table 5). For patients with malignant melanoma, the odds ratio (OR) for the occurrence of nAE was 0.286 (CI: 0.1–0.6, *p* < 0.001). Patients receiving additional chemotherapy were approximately five times more likely to develop nAE (OR = 5.297, CI: 1.7–16.4, *p* = 0.004), and patients in a palliative setting exhibited an OR of 2.301 (*p* = 0.023) compared to patients in an adjuvant setting.

**Table 5:** Illustration of the statistically significant results of the patient and treatment characteristics when comparing the two subgroups with and without neurotoxicity.

Parameters	Subgroup Comparison, *p*-Value	Univariate Statistics, OR (CI), *p*-Value	Multivariate Statistics, OR (CI), *p*-Value
Malignant melanoma vs. other tumor diseases	<0.001	0.286 (0.1–0.6), <0.001	0.294 (0.1–0.7), 0.006
PD-(L)-1-Inhib. vs. PD-(L)-1-Inhib. + chemotherapy	0.002	5.297 (1.7–16.4), 0.004	5.256 (1.7–16.3), 0.004
Age at first diagnosis, years	0.098	0.979 (0.955–1.004), 0.100	0.967 (0.939–0.997), 0.031
Palliative vs. adjuvant setting	0.021	2.301 (1.1–4.7), 0.023	/

Note: OR: odds ratio, PD-1: programmed cell death protein 1.

**Figure 2:**
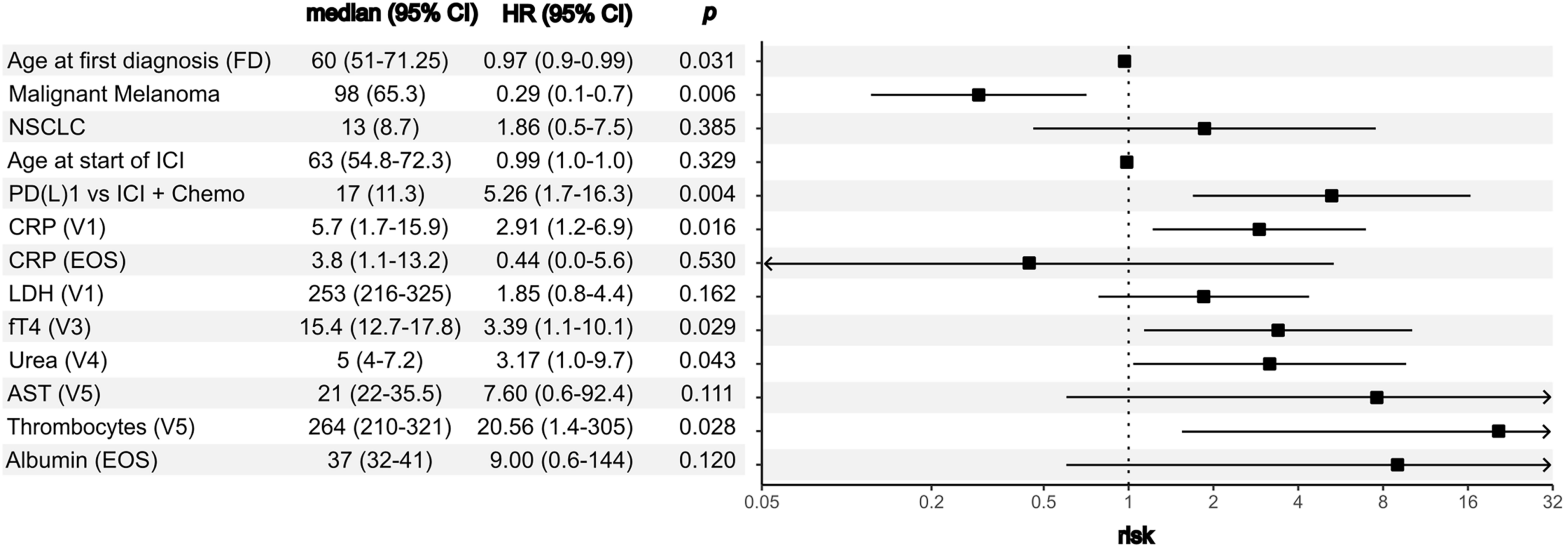
Multivariate risk factor analysis for the development of neurological adverse events. CI: confidence interval, HR: hazard ratio, NSCLC: non-small cell lung cancer, ICI: immune checkpoint inhibitors, PD-1: programmed cell death protein 1, CRP: C-reactive protein; LDH: lactate dehydrogenase, fT4: free thyroxine, AST: aspartate aminotransferase.

Multivariate analysis (Table 5, Fig. 2) confirmed malignant melanoma as an independent protective factor (OR = 0.294, CI: 0.1–0.7, *p* = 0.006), indicating that patients with malignant melanoma in this cohort were significantly less likely to develop nAE. In contrast, combination therapy with a PD-(L)1 inhibitor and chemotherapy was identified as an independent risk factor compared with ICI monotherapy (OR = 5.256, CI: 1.7–16.3, *p* = 0.004) (Table 5, Fig. 2).

Regarding laboratory parameters, the univariate analysis identified seven potential risk factors for nAE at different time points (*p* < 0.2) when applying the cohort median as the threshold. Except for bilirubin, elevated levels of all markers were associated with an increased likelihood of nAE. In the multivariate analysis, four of these seven parameters remained significant (*p* < 0.05) (Table 6). The most notable association was observed for elevated CRP levels at follow-up 1 (3–4 weeks after ICI initiation), which significantly increased the risk of nAE (OR = 2.909, CI: 1.2–6.9, *p* = 0.016) (Fig. 2). Elevated fT4 levels at follow-up 3 (OR = 3.394, CI: 1.1–10.1, *p* = 0.029), urea levels at follow-up 4 (OR = 3.167, CI: 1.0–9.7, *p* = 0.043) and platelet count at follow-up 5 (OR = 20.557, CI: 1.4–305, *p* = 0.028) were likewise associated with an increased risk of nAE (Table 6, Fig. 2).

**Table 6:** Illustration of laboratory parameters that differed significantly between patients with and without neurotoxicity.

Time	Parameter (Reference Values, MHH Laboratory)	Median Total Cohort	Subgroup Comparison, *p*-Value	Univariate Statistics, OR (CI), *p*-Value	Multivariate Statistics, OR (CI), *p*-Value
Baseline	sIL2R (223–710 kU/L)	651 kU/L	0.056	2.699 (1.0–7.1), 0.045	/
FU1 (3–4 weeks)	CRP (≤5 mg/L)	4.2 mg/L	0.041	3.222 (1.5–7.2), 0.004	2.909 (1.2–6.9), 0.016
	Bilirubin (2–21 mmol/L)	6 mmol/L	0.080	2.261 (1.0–5.0), 0.043	/
FU3 (9–12 weeks)	fT4 (12–22 pmol/L)	14.4 pmol/L	0.034	2.806 (1.2–6.6), 0.018	3.394 (1.1–10.1), 0.029
FU4 (3–4 months)	Urea levels (2.8–7.2 mmol/L)	5 mmol/L	0.085	3.167 (1.0–9.7), 0.043	3.167 (1.0–9.7), 0.043
FU5 (4–5 months)	Thrombocytes (150–370 tsd/µL)	264 tsd/µL	0.096	2.667 (0.9–8.4), 0.093	20.557 (1.4–305), 0.028
	AST (≤35 U/L)	26 U/L	0.019	6.750 (1.9–23.4), 0.003	7.600 (0.6–92.4), 0.111

Note: AST: aspartate aminotransferase; CRP: C-reactive protein; fT4: free thyroxine; FU: follow up; OR: Odds ratio; sIL2R: soluble interleukin-2 receptor.

## 4 Discussion

In this study, we aimed to gain a deeper understanding of nAE in ICI-treated patients. We also addressed the question of potential risk factors and potential laboratory biomarkers for nAE, which have not yet been identified [20–23]. The strength of this report lies in the size of the ICOG registry and the prospective neurological evaluation of ICI-treated patients, with a specific focus on nAE. All patients underwent a comprehensive and systematic neurological assessment, which likely contributed to the comparatively high incidence of nAE observed in this study (36.7%, n = 55). This rate exceeds those reported in earlier, predominantly retrospective studies (2%–18%) [17,24,25]. Even after excluding less specific symptoms such as paresthesia and myalgia, the frequency remained at approximately 19%, suggesting that nAE may be more common than previously assumed. However, the incidence of severe nAE (CTCAE grade ≥ 3) was only slightly higher than in existing literature [26], suggesting that the increased overall incidence in our cohort was largely driven by milder symptoms, which can often go undetected in routine clinical care. It should therefore be noted that the broad symptom recording and inclusive definition of nAE employed in this study must be considered when interpreting the results.

While most nAE were mild and involved the peripheral nervous system, we also observed severe manifestations, such as chronic inflammatory demyelinating polyneuropathy (CIDP, CTCAE III) and pronounced myalgia (CTCAE III), both of which substantially impair activities of daily living. Central nervous system involvement occurred less frequently, with single cases of autoimmune encephalitis, cerebral vasculitis, and absence-like episodes. Overall, severe nAE (CTCAE grade ≥ III) were identified in 3.3% of all patients, accounting for 9.1% of all nAE, indicating a slightly higher incidence than previously reported [26].

The relevance of neurological adverse events is underscored not only by their substantial impact on patients, but also by their consequences for cancer treatment. In this cohort, neurotoxicity led to treatment interruptions (1.3%, n = 2), permanent discontinuations (2.7%, n = 4), and hospitalisations (2.7% of all patients and 7.3% of those with nAE). 12.67% of all patients in the cohort required further diagnostic tests due to actual or suspected nAE. Although MRI examination is an important diagnostic measure, only a minority of patients (2% of all patients and 5.5% of those with nAE) underwent cerebral or spinal MRI. This reflects the fact that MRI was reserved for cases with a clear clinical indication, typically patients presenting with focal deficits or CTCAE ≥ 3, which explains the low frequency of MRI despite systematic neurological assessments.

Given the clinical relevance of long-term neurological impairment, we next examined recovery rates in our cohort. Interpretation of these outcomes is limited by the relatively short observation window, the late onset of many nAE, and substantial loss to follow-up. Notably, patients who recovered or showed clinical improvement developed symptoms earlier (median: 21 days) than those who did not recover (median: 90 days), which may have resulted in shorter effective follow-up periods for some individuals. These factors may underestimate or misclassify long-term outcomes, and conclusions regarding sustained neurological recovery should therefore be drawn with caution. Despite these limitations, a trend toward persistent neurological symptoms emerged. Only 23.6% of patients with nAE achieved full recovery by the end of the study period, 4.7% showed improvement, and 41.8% exhibited no improvement. These findings align with previous reports indicating that ICI-associated neurological symptoms frequently follow a chronic course, with lasting effects on quality of life and overall prognosis [27,28].

The results reinforce our initial hypothesis that nAE poses a significant risk, making early diagnosis and intervention critical.

Statistical analyses identified two baseline patient characteristics and four laboratory parameters that were associated with the occurrence of nAE. Unpaired *t*-tests and univariate binary logistic regressions revealed an association between ICI-induced neurotoxicity and factors such as the type of underlying tumour (specifically malignant melanoma), concurrent chemotherapy and a palliative setting.

Further multivariate analyses confirmed an association between malignant melanoma as the underlying tumor entity and a lower likelihood of developing nAE. At first glance, this suggests that patients with malignant melanoma are significantly less prone to nAE. However, this observation contrasts with previous reports, which have generally identified malignant melanoma, particularly due to the frequent use of dual ICI regimens, as a risk factor for neurological toxicity [21,22]. One possible explanation for this discrepancy is the high proportion of melanoma patients in our cohort, which may introduce selection bias as a confounding factor. In our cohort, most melanoma patients were in an adjuvant setting and thus received monotherapy with PD-1 inhibitors more frequently than immune doublet therapy (77.6% vs. 22.4%), whereas prior studies frequently included higher proportions of dual ICI therapy or patients with advanced-stage disease. Since monotherapy with a single ICI is associated with a lower risk of nAE [7,21], these treatment and sampling differences may account for the lower risk observed.

Concurrent chemotherapy was found to increase the likelihood of patients developing nAE by fivefold in a multivariate analysis. However, this association should be interpreted with caution, given that cytotoxic agents can induce peripheral neurotoxicity themselves [29,30]. This confounding effect is inherent in real-world treatment settings and may partially explain the observed association.

Similarly, univariate analyses showed an association between a palliative treatment setting and an increased risk of nAE, which is plausibly explained by the more frequent administration of chemotherapy alongside ICI in this group. Because the variable “palliative vs. adjuvant setting” was collinear with concomitant chemotherapy, it could not be included in the multivariate analysis.

The observation aligns with recent studies suggesting that prior or concomitant chemotherapy may predispose patients to ICI-related neurotoxicity, although these findings stem largely from retrospective analyses [21,31].

Univariate analysis of laboratory test results revealed an association between the development of nAE and seven potential factors, including elevated levels of sIL2R, CRP, fT4, urea, thrombocytes and AST at various time points, as well as decreased bilirubin levels 3–4 weeks after the initiation of ICI treatment. Multivariate analysis confirmed this association for elevated CRP (at 3–4 weeks), fT4 (at 9–12 weeks) and urea (at 3–4 months). Additionally, elevated platelet levels were noted 4–5 months post-ICI, despite only passing the threshold (*p* < 0.2) in the univariate findings, rather than reaching statistical significance (*p* < 0.05).

From a pathomechanistic perspective, CRP, sIL2R, and platelet levels stand out as promising markers for monitoring autoimmune adverse events. The association between elevated CRP levels and nAE is of particular interest and is supported by previous studies [32–35]. Mechanistically, early CRP elevation may reflect systemic immune activation triggered by ICI therapy. Increased T-cell activation and cytokine release can alter endothelial function, potentially increasing blood–brain barrier permeability and facilitating neuroinflammatory processes in susceptible individuals on a transient basis [36,37]. Although CRP is not a neuro-specific marker, an increase in its levels could potentially serve as an accessible surrogate for these upstream inflammatory pathways, potentially preceding overt nAE. In our cohort, CRP elevation above the median at 3–4 weeks after treatment initiation may represent an early indicator of subsequent nAE, which typically occurred around week 6 (Supplementary Fig. S1). This suggests a potentially valuable window for closer monitoring and early neurological assessment. The observation is consistent with recent studies demonstrating an association between elevated CRP levels and the development of irAEs [34,38,39]. Accordingly, patients receiving combined chemotherapy and ICI who exhibit an early rise in CRP might benefit from timely neurological evaluation to avoid missing early signs of ICI-associated neurotoxicity.

While our study focused on standard laboratory parameters, emerging evidence highlights the potential predictive value of additional soluble factors, such as cytokines [39–42]. Although these markers may offer greater specificity, their use in routine clinical practice is limited by availability and logistical constraints. In contrast, the laboratory parameters assessed in our study are widely accessible and easily integrated into standard oncological monitoring.

It is important to acknowledge the limitations of this cohort study. The reported incidence of nAE may not be directly comparable to that in previous studies, since this analysis employed a deliberately broad definition of nAE. This approach was chosen in order to capture the full spectrum of neurological manifestations, improve early detection, and avoid underreporting. However, due to this broad definition, incidence estimates may be higher than those reported in cohorts with a narrower definition. A sensitivity check that excluded non-specific symptoms (e.g., paresthesia and myalgia) still yielded an incidence of approximately 19%, indicating that broader screening likely captures clinically relevant events. As the subgroup of severe nAE was small and clinically heterogeneous, CTCAE-stratified analyses were not feasible, but this should be highlighted as an important objective for future studies.

Additionally, while the wide selection of laboratory parameters provided valuable exploratory insights, their non-specific nature means they may have been influenced by comorbidities or unrelated systemic factors. Given the exploratory nature of this study and the large number of laboratory parameters assessed at multiple time points, no formal correction for multiple comparisons was applied. Consequently, the risk of type I error is increased, and the findings should be interpreted as hypothesis-generating. Furthermore, the use of median-based dichotomisation is a pragmatic exploratory approach, but it inherently reduces information and may obscure dose–response relationships. Future studies with larger datasets should apply continuous modelling or clinically defined cutoffs, such as ROC-based thresholds.

Lastly, although an elevation in CRP at 3–4 weeks was statistically associated with the development of nAE later on, the considerable overlap between groups limits its clinical discriminatory value. Therefore, CRP should be interpreted as a non-specific early warning signal rather than a predictive biomarker. Due to the small number of cases of severe nAE and the clinical heterogeneity of the cohort, it was not feasible to conduct severity-stratified biomarker analyses, which should be addressed in future studies with larger sample sizes. Future research should also aim to establish clinically meaningful thresholds and improve marker specificity.

In conclusion, nAE associated with ICI therapy pose a significant risk as they may occur more frequently, and potentially affect quality of life more substantially than previously estimated. This study identified an association between nAE and the aforementioned variables, particularly elevated CRP 3–4 weeks after the start of ICI treatment. Patients undergoing ICI treatment could be assessed for these potential risk factors, with high-risk groups monitored closely for early signs of nAE. Routine blood tests could include tracking key markers, such as CRP levels, early in treatment.

Our findings suggest that elevated CRP levels at 3–4 weeks after the start of ICI treatment may indicate an increased risk of neurotoxicity. Although CRP lacks specificity, an increase above customary thresholds (>5 mg/L) or above the patient’s baseline should prompt heightened clinical vigilance. This is particularly important for patients receiving concomitant chemotherapy or exhibiting other identified risk factors, as early neurological consultation can support the timely diagnosis and management of nAE.

Further research is essential to refine and confirm these markers with the ultimate goal of enabling an earlier identification of patients at risk of nAE and facilitating timely intervention to reduce the number of patients affected by potentially long-lasting neurotoxicity.

## Supplementary Materials



## Data Availability

The data that support the findings of this study are available from the Corresponding Author, Laura Duzzi, upon reasonable request.

## Supplementary Materials

The supplementary material is available online at https://www.techscience.com/doi/10.32604/or.2026.074095/s1.
