# Supplementary material for: Elevated C-Reactive Protein as a Potential Biomarker for Neurological Adverse Events in Immune Checkpoint Inhibitor Therapy: A Prospective Cohort Study
Source: Oncol Res. 2026 Apr 22;34(5):17. doi: 10.32604/or.2026.074095 (PMC13126388; doi:10.32604/or.2026.074095)
Supplement: Supplementary file 1 [file OncolRes-34-74095-s001.docx]

**Supplementary Table S1:** Study overview.

| Characteristic of each study visit | Baseline | Follow up 1 | Follow up 2 | Follow up 3 | Follow up 4 | Follow up 5 | End of Study |
| --- | --- | --- | --- | --- | --- | --- | --- |
| Time | Before start of ICI | 3-4 weeks | 6-8 weeks | 9-12 weeks | 3-4 months | 4-5 months | 6 months |
| Blood samples | x | x | x | x | x | x | x |
| Interview regarding nAE | x | x | x | x | x | x | x |
| Clinical scores | x | x | x | x | x | x | x |
| Neurological examination | x |  |  |  |  |  | x |

Standard procedures included biosampling, collecting clinical scores (INCAT, RODS, Karnofsky-Score, ECOG) and obtaining medical history. At baseline and EOS, a detailed neurological examination was performed in addition. ICI: immune checkpoint inhibitors, nAE: neurological adverse events. Neurological examination: performed by subinvestigator particularly trained in clinical neurological assessment. RODS: Rasch-built Overall Disability Scale. INCAT: Inflammatory Neuropathy Cause and Treatment disability score. ECOG: Eastern Cooperative Oncology Group status.

**Supplementary Table S2:** Serum parameters with significant differences between the nAE and non-nAE subgroups at different time points (unpaired t test).

| Parameter (unit), point in time | **Total cohort**  **(n = 150),**  **mean (range)** | **Non-nAE-subgroup (n = 95),**  **mean (range)** | **nAE-subgroup**  **(n = 55),**  **mean (range)** | ***p*-value** |
| --- | --- | --- | --- | --- |
| Potassium (mmol/L),  FU3 | 4.5 (3.4-5.5) | 4.5 (3.4-5) | 4.5 (3.7-5.5) | 0.046 |
| CRP (mg/L) |  |  |  |  |
| FU1 | 5.7 (0.2-188.9) | 2.7 (0.5-141.3) | 8.9 (0.2-188.9) | 0.041 |
| EOS | 3.8 (0.6-215.3) | 2.6 (0.6-161.6) | 4.5 (0.6-215.3) | 0.026 |
| AST (U/L),  FU5 | 26 (14-117) | 24 (16-46) | 30.5 (14-117) | 0.019 |
| ALT (U/L),  FU2 | 23.5 (6-105) | 23 (6-59) | 24 (10-105) | 0.027 |
| Ferritin (µg/L),  EOS | 116 (23-1777) | 100 (23-755) | 139 (33-1777) | 0.040 |
| Thrombocytes (tsd/µL),  FU4 | 264 (74-616) | 301 (136-616) | 227 (74-438) | 0.024 |
| fT4 (pmol/L),  FU3 | 15.4 (1.3-43.8) | 14.4 (4.4-28.7) | 16.7 (1.3-43.8) | 0.034 |

ALT: alanine aminotransferase; AST: aspartate aminotransferase; CRP: C-reactive protein; fT4: free thyroxine; nAE: neurological adverse events. FU1: follow-up 1 (3-4 weeks), FU2: follow-up 2 (6-8 weeks), FU3: follow-up 3 (9-12 weeks), FU4: follow-up 4 (3-4 months), FU5: follow-up 5 (4-5 months), EOS: end of study, last follow-up (6 months after start of ICI).


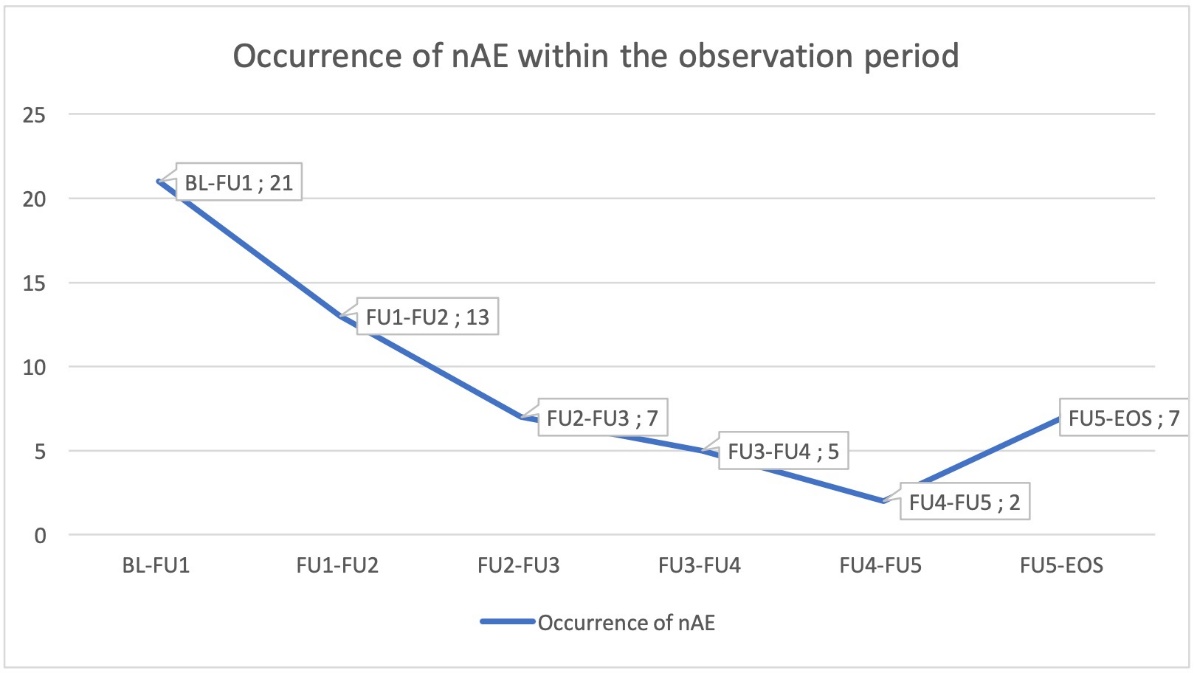


**Supplementary Figure S1:** Occurrence of nAE across follow-up intervals (x-axis: time intervals; y-axis: number of nAE).Most nAE emerged early after initiation of ICI therapy, with a progressive decline over subsequent follow-ups. BL = baseline (therapy start); FU1 = 3–4 weeks; FU2 = 6–8 weeks; FU3 = 9–12 weeks; FU4 = 3–4 months; FU5 = 4–5 months; EOS = end of study (6 months after therapy initiation).
